# New Insights into the Methylation of *Mycobacterium tuberculosis* Heparin Binding Hemagglutinin Adhesin Expressed in *Rhodococcus erythropolis*

**DOI:** 10.3390/pathogens10091139

**Published:** 2021-09-04

**Authors:** Cristina Parada, Isabel Cecilia Neri-Badillo, Antonio J. Vallecillo, Erika Segura, Mayra Silva-Miranda, Silvia Laura Guzmán-Gutiérrez, Paola A. Ortega, Enrique Wenceslao Coronado-Aceves, Laura Cancino-Villeda, Alfredo Torres-Larios, Michel de Jesús Aceves Sánchez, Mario Alberto Flores Valdez, Clara Espitia

**Affiliations:** 1 Departamento de Inmunología, Instituto de Investigaciones Biomédicas, Universidad Nacional Autónoma de México, Ciudad de México 04510, Mexico; mariac@iibiomedicas.unam.mx (C.P.); cecineribadillo@hotmail.com (I.C.N.-B.); antonio.vallecillo@ucuenca.edu.ec (A.J.V.); eseguras@iibiomedicas.unam.mx (E.S.); msilvami@conacyt.mx (M.S.-M.); laura.guzman@iibiomedicas.unam.mx (S.L.G.-G.); paoandortega@hotmail.com (P.A.O.); wenceslao.coronadoa@iibiomedicas.unam.mx (E.W.C.-A.); anacv1009@gmail.com (L.C.-V.); 2 Facultad de Ciencias Agropecuarias, Universidad de Cuenca, Cuenca 010220, Ecuador; 3 Consejo Nacional de Ciencia y Tecnología, CONACyT, Ciudad de México 03940, Mexico; 4 Department of Biochemistry and Structural Biology, Instituto de Fisiología Celular, Universidad Nacional Autónoma de México, Ciudad de México 04510, Mexico; torres@ifc.unam.mx; 5 Biotecnología Médica y Farmacéutica, Centro de Investigación y Asistencia en Tecnología y Diseño del Estado de Jalisco, A.C., Guadalajara 44270, Mexico; mj.aceves.s@gmail.com (M.d.J.A.S.); floresv@ciatej.mx (M.A.F.V.)

**Keywords:** *Mycobacterium tuberculosis*, recombinant HbhA, *Rhodococcus erythropolis* methylation

## Abstract

In recent years, knowledge of the role that protein methylation is playing on the physiopathogenesis of bacteria has grown. In *Mycobacterium tuberculosis*, methylation of the heparin binding hemagglutinin adhesin modulates the immune response, making this protein a subunit vaccine candidate. Through its C-terminal lysine-rich domain, this surface antigen interacts with heparan sulfate proteoglycans present in non-phagocytic cells, leading to extrapulmonary dissemination of the pathogen. In this study, the adhesin was expressed as a recombinant methylated protein in *Rhodococcus erythropolis* L88 and it was found associated to lipid droplets when bacteria were grown under nitrogen limitation. In order to delve into the role methylation could have in host–pathogen interactions, a comparative analysis was carried out between methylated and unmethylated protein produced in *Escherichia coli*. We found that methylation had an impact on lowering protein isoelectric point, but no differences between the proteins were found in their capacity to interact with heparin and A549 epithelial cells. An important finding was that HbhA is a Fatty Acid Binding Protein and differences in the conformational stability of the protein in complex with the fatty acid were observed between methylated and unmethylated protein. Together, these results suggest that the described role for this mycobacteria protein in lipid bodies formation could be related to its capacity to transport fatty acids. Obtained results also provide new clues about the role HbhA methylation could have in tuberculosis and point out the importance of having heterologous expression systems to obtain modified proteins.

## 1. Introduction

Methylation is a common post-translation modification, occurring in a wide range of prokaryotic and eukaryotic proteins, e.g., cytochrome *c*, histones, actin, myosin, myelin, flagella proteins, ribosomal proteins, heterogeneous nuclear ribonucleoproteins and translation factors [1]. Methylation of non-histone prokaryotic proteins modulates diverse functions. For instance, pilin methylation of *Synechocystis* sp. regulates cell motility and methylation of EF-Tu factor in *Escherichia coli* decreases its GTPase activity, stimulating its dissociation from membrane [2]. Furthermore, trimethylated lysine residues of outer membrane protein B (OmpB) of *Rickettsia typhi* are associated with virulence, while protein from non-virulent strains contains mainly monomethyl lysine. In addition, methylation of OmpB is involved in immunogenicity in all rickettsia species, serving as a protective envelope that mediates host cell adhesion and invasion [3,4].

Two adhesins of *M. tuberculosis*, the heparin binding hemagglutinin (HbhA, Rv0475) and the histone-like protein (Hlp/HupB, Rv2986), are molecules modified by methylation. At least 13 out of 16 lysine residues in the C-terminus of *M. tuberculosis* HbhA were shown to be mono or dimethylated [5,6], while in HupB, five lysine and two arginine residues are modified by acetylation or methylation [7]. On the other hand, methylation of HbhA is associated with the induction of a protective T cell immune response in mice [8]. In humans, a higher IFN-γ response to methylated protein was observed in individuals with latent tuberculosis, compared to the response of patients with active tuberculosis [9]. Even more, there is evidence that a methylated HbhA T cell peptide epitope is recognized by T cells from humans latently infected with *M. tuberculosis* as was evidenced by IFN-γ release upon peptide stimulation. The unmethylated peptide did not induce IFN-γ, arguing that the methyl lysine is part of the T cell epitope [10]. HbhA is also involved in the extrapulmonary dissemination of mycobacteria [11]. The protein interacts with epithelial cells through Syndecans, a protein family of four members (Sdc1–Sdc4) that possesses domains covalently attached to heparan sulfate chains [12]. Binding of HbhA to proteoglycans is mediated by the lysine-rich C-terminal, which contains the domain where methylation takes place, but the role of lysine methylation in this interaction is not completely understood.

In this study, *R. erythropolis,* a bacterium that belongs to a genus genetically closely related to *M. tuberculosis* [13], was used as a surrogate host to express *M. tuberculosis* HbhA. We were prompted to use this system due to the identification of an HbhA orthologous in *Rhodococcus opacus* PD630, the triacylglycerol accumulation deficient protein (TadA) involved in the assembly and maturation of lipid droplets (LD) [14]. Later, it was demonstrated that through this protein, LD *Rhodococcus jostii* RHA1 bind to genomic DNA, increasing the survival rate of bacteria under nutritional and genotoxic stress [15]. More recently, the participation of mycobacteria HbhA in LD formation was demonstrated, as well as the capacity of HbhA to specifically binds to a eukaryotic lipid, 4,5 di-phosphorylated phosphatidylinositol [16]. Here, we report that recombinant HbhA expressed in *R. erythropolis* was methylated, and importantly, as native mycobacterial HbhA, the recombinant in *R. erythropolis* was found associated to LD induced when recombinant bacteria were grown under nitrogen deprivation. Differences between methylated HbhA expressed in *R. erythropolis* and unmethylated expressed in *E. coli* were found, with regard to their isoelectric point (pI) and in their capacity to bind fatty acids (FA). Small differences in interaction of both methylated and unmethylated proteins with proteoglycans and with A549 epithelial cells were found. Together, the results contribute to our understanding of the putative role that methylation of HbhA could have in *M. tuberculosis.* This work points out *R. erythropolis* as a surrogate bacterium to express methylated *M. tuberculosis* proteins, providing a system to study their putative physiopathological roles.

## 2. Results

### 2.1. Expression and Purification of M. tuberculosis HbhA from Recombinant R. erythropolis

**Figure 1:**
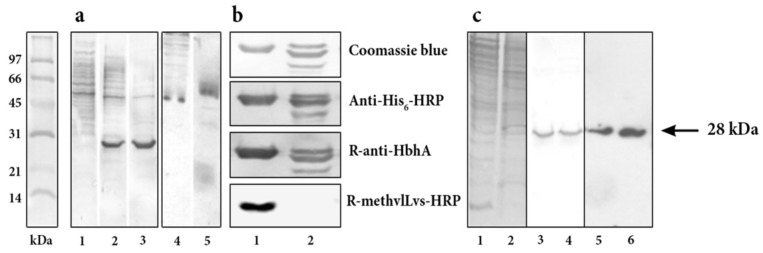
Expression of r*Rho*HbhA from *R. erythropolis* transformed with pTip-QC1-HbhA vector. (**a**) Coomassie blue staining of whole sonicated extract proteins from *R. erythropolis* L88 transformed with pTip QC1vector without insert (lane 1). Whole sonicated extract and insoluble fraction of *R. erythropolis* transformed with pTip-QC1-*hbha* (lanes 2 and 3). Negative controls: Western blot of whole sonicated extract from *R. erythropolis* transformed with pTip-QC1 without insert, incubated with rabbit-anti-HbhA and anti-His_6_-HRP antibodies (lanes 4 and 5, respectively). (**b**) Purified r*Rho*HbhA (lane 1) and r*E.coli*HbhA (lane 2). Proteins were resolved by PAGE-SDS and stained with Coomassie blue. By Western blot, both proteins were detected with anti-His_6_-HRP and R-anti-HbhA antibodies, but only r*Rho*HbhA was recognized by R-anti-methylLys-HRP. (**c**) Coomassie blue staining of soluble extract and cell wall proteins from r*BCG*HbhA transformed with pMV361 vector (lanes 1 and 2). Soluble extract and cell wall from r*BCG*HbhA proteins were detected by Western blot with R-anti-HbhA (lanes 3 and 4) and with R-anti-methylLys-HRP (lanes 5 and 6). Recombinant HbhA is indicated with an arrow.

*R. erythropolis* L88 grown in Luria Bertani medium supplemented with Chloramphenicol (LB/Chl) was transformed with pTip-QC1 with and without *hbha* insert. Recombinant extracts and purified recombinant HbhA were resolved by SDS-PAGE and transferred to PDVF membranes. Coomassie blue staining of whole sonicated extract (WSE) and insoluble fraction (IF) from transformed bacteria are shown in Figure 1a. As expected, no expression of r*Rho*HbhA was observed in the control without insert, in contrast with bands of ~28 kDa present in WSE and IF from bacteria transformed with the plasmid with insert (lanes 2 and 3, respectively). Unspecific reactivity was observed by Western blot (WB), with both rabbit anti-HbhA polyclonal antibody (R-anti-HbhA) and with anti-6xHis-tag-horseradish peroxidase (HRP) antibody (anti-His_6_-HRP) in WSE from negative control without insert.

*M. tuberculosis* HbhA was also expressed in *M. bovis* BCG. Soluble extract (SE) and cell wall (CW) proteins were resolved by SDS-PAGE and transferred to PDVF membranes. Coomassie blue staining of WSE and SE are shown in Figure 1c. HbhA proteins were recognized by WB, with R-anti-HbhA. The presence of methyl groups was evidenced in both WSE and SE with R-anti-methylLys-HRP.

### 2.2. Molecular Mass Determination

By mass spectrometry of r*Rho*HbhA, a major peak with a MM of 22,828 Da was observed. Removing the six-histidine tag and amino acids prom linker peptide adding by plasmid sequences, the MM should be 21,817.091 Da, which is very close to the 21,796 Da derived of mass spectrometry analysis of purified native HbhA. Comparing with the theoretical mass of the native protein of 21,475.39 Da, the estimated difference of 341 Da would correspond to 24.4 methyl groups, very similar to the value previously reported for methylated native *M. tuberculosis* HbhA [6].

### 2.3. Isolation and Characterization of Lipid Droplets Induced in Recombinant R. erytropolis Strain Grown in M9 Medium with Low Nitrogen and Carbon Excess

*R. erythropolis* recombinant strains r*Rho*HbhA, r*Rho*PstS1 and r*Rho*Apa were grown under nitrogen starvation conditions. The IF from those strains were obtained to confirm the expression of recombinant proteins from bacteria grown under this condition. 

**Figure 2:**
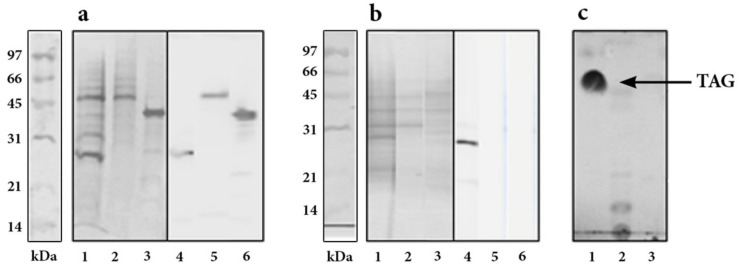
Expression of *M. tuberculosis* HbhA, Apa and PstS-1 in *R. erythropolis* grown in M9GhiNlow (**a**) Coomassie blue staining of IF from r*Rho*HbhA (lane 1), r*Rho*Apa (lane 2) and r*Rho*PstS-1 (lane 3). WB of the same proteins detected with anti-His6-HRP (lanes 4, 5 and 6). (**b**) Coomassie blue staining of proteins extracted from LD purified from r*Rho*HbhA (lane 1), r*Rho*Apa (lane 2) and r*Rho*PstS-1 (lane 3). WB of the same proteins detected with anti-His6-HRP (lanes 4, 5 and 6). (**c**) TLC of LD extracted from r*Rho*HbhA strain, an arrow (line 2) indicates TAG. Glyceryl trioleate and glyceryl tripalmitate used as standards are shown in lane 1 and 3, respectively.

Coomassie blue staining of IF extracts from recombinant strains resolved by SDS-PAGE, and recombinant proteins from the same extracts recognized by anti-His_6_-HRP by WB are shown in Figure 2a.

Growing recombinant strains in M9 low nitrogen and carbon excess medium, supplemented with 4% glucose and 0.15% of NH_4_SO_2_. (M9G_Hi_N_Low_), induced the production of LD, which were purified by a sucrose gradient and treated to eliminate unspecific bound proteins. Lipids present in LD were solubilized and proteins were precipitated and separated by SDS-PAGE. Several protein bands from LD of recombinant r*Rho*HbhA, r*Rho*Psts, and r*Rho*Apa between 50 and 25 kDa were observed by Coomassie blue-staining (Figure 2b). After the extensive treatment to eliminate unspecific bound proteins, only r*Rho*HbhA was recognized by anti-His_6_-HRP by WB, indicating that the binding of HbhA to LD was highly specific (Figure 2b).

Solubilized lipids present in LD were separated by thin-layer chromatography (TLC) (Figure 2c). This fraction contains triacylglycerol (indicated by an arrow), diacylglycerol, and free FA that occurred with other components (lane 2). Glyceryl trioleate and glyceryl tripalmitate were used as standards. It is worth pointing out that LD were only found in both wild-type and recombinant strains but only when bacteria were grown under nitrogen starvation conditions.

### 2.4. Characterization of Recombinant Proteins by 2-DE

**Figure 3:**
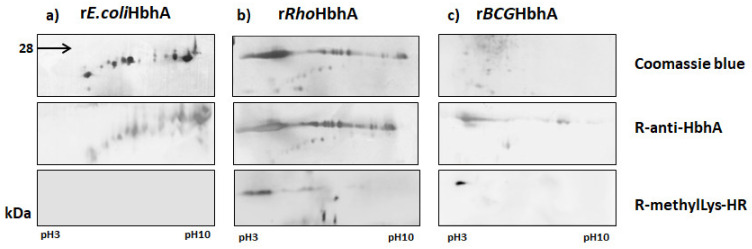
Two-dimensional electrophoresis and SDS-PAGE. Recombinant purified r*E.coli*HbhA (**a**), r*Rho*HbhA (**b**) and SE from r*BCG*HbhA (**c**) are shown in each column. The first row corresponds to Coomassie blue staining of the proteins, the second and third row show the recognition of the spots with R-anti-HbhA R-anti-methylLys-HRP antibodies by WB.

To study the pI pattern, purified r*E.coli*HbhA, r*Rho*HbhA and SE from r*BCG*HbhA were resolved by 2-DE in pH range between 3 to 10. Coomassie blue staining of r*Ecoli*HbhA showed several spots concentrated in the basic pH region, many of them with lower molecular weights that migrated along the pH gradient, probably due to protein degradation. All the spots were recognized by R-anti-HbhA, but not by R-anti-methylLys-HRP, confirming the absence of methylation of rHbHA expressed in *E. coli* (Figure 3a). 

In contrast, when purified r*Rho*HbhA was resolved by 2-DE, spots were preferentially concentrated in the pH acid region, as shown by Coomassie blue staining. R-anti-HbhA recognized all the spots by WB, but R-anti-methylLys-HRP only recognized the spots with low pI, located in the acid pH region (Figure 3b).

Since it is known that *M. tuberculosis* HbhA expressed in *M. bovis* BCG is methylated, we also studied the 2-DE pattern of the protein from SE of recombinant strain as a positive control of protein methylation. By WB, R-anti-HbhA recognized spots in both the acid and basic pH zone. However, R-anti-methylLys-HRP only recognized the spots located in the acid pH zone, confirming that methylated r*BCG*HbhA also showed a low pI (Figure 3c).

### 2.5. Binding of rE.coliHbhA and rRhoHbhA to Heparin

The measure of affinity of biotinylated heparin for recombinant proteins r*E.coli*HbhA and r*Rho*HbhA by biolayer interferometry was performance.

**Figure 4:**
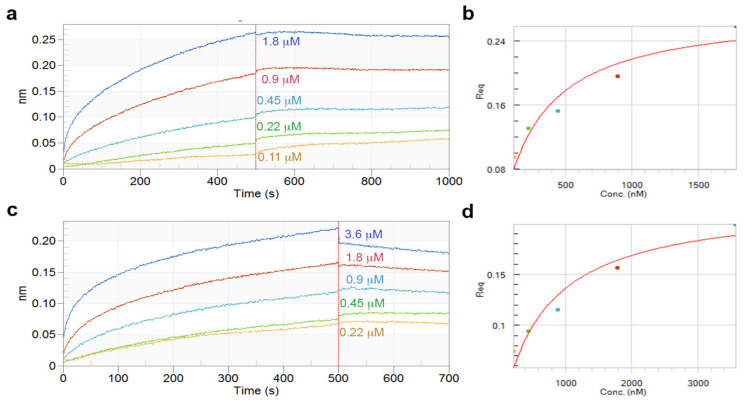
Real-time biolayer interferometry sensorgrams for determination of binding affinity (**a**,**c**) Sensorgrams showing binding on streptavidin biosensors of biotinylated heparin with increased concentrations of recombinants HbhA. (**b**,**c**) Steady-state analysis of the binding response (nm) as a function of the concentration of HhbA. Calculated K*_D_* = 0.28 μM (R2 = 0.92) and 0.62 μM (R2 = 0.95) for the non-methylated r*E.coli*HbhA (**a**,**b**) and methylated r*Rho*HbhA (**c**,**d**).

Recombinants HbhA interacted with heparin in a concentration-dependent manner and allowed us to calculate a dissociation constant value of 0.28 μM and 0.62 μM for the unmethylated r*E.coli*HbhA and methylated r*Rho*HbhA, respectively (Figure 4).

Two reference controls were used, one that did not include the protein, only biotinylated heparin, and another that used biotinylated BSA instead of biotinylated heparin. No binding of biotinylated heparin to the biosensor or binding to biotinylated BSA to HbhA was detected.

**Figure 5:**
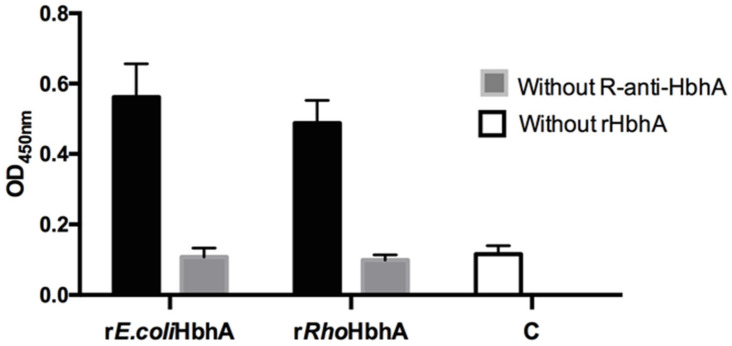
ELISA ligand assay. Interaction of recombinant r*E.coli*HbhA and r*Rho*HbhA with biotinylated heparin bound to streptavidin-coated plates. Binding was detected with R-anti-HbhA. Black bars represent the interaction signal towards heparin. Gray bars are controls without R-anti-HbhA and bar c indicates the control without rHbhA protein. The results represent 2 independent experiments. The ANOVA and Tukey’s statistical analysis did not show significant differences.

The specificity of r*E.coli*HbhA and r*Rho*HbhA to heparin was also studied by an enzyme-linked immunosorbent assay (ELISA). The results are shown in Figure 5, with both unmethylated r*E.coli*HbhA and methylated r*Rho*HbhA (black bars) bound to heparin. 

It is important to mention that although a higher reactivity with heparin of methylated r*Rho*HbhA was consistently observed in all the experiments we carried out, no significant differences were found.

### 2.6. Binding of rE.coli HbhA and rRhoHbhA to A549 Cells and Post-Infection of Cells with BCG

**Figure 6:**
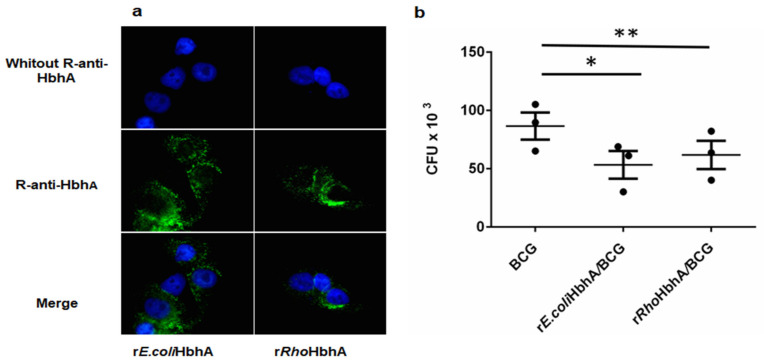
Immunofluorescence microscopy analysis. (**a**) Binding of purified r*Rho*HbhA and r*E.coli* HbhA to human A549 cells. Bound proteins were detected using a R-anti-rHbhA followed by incubation with anti-IgG coupled to Alexa Fluor 488 as secondary antibody. As negative control, cells without R-anti-HbhA were used. More than 200 fields were examined for each condition using the 100× magnification by fluorescence microscopy. (**b**) Infection of A549 cells previously incubated with r*E.coli*HbhA and r*Rho*HbhA and then with *M. bovis* BCG; as negative control, the R-anti-rHbhA was omitted. Binding Data represent three independent experiments; asterisks describe statical significance differences between *M. bovis* BCG control vs *M. bovis* BCG/r*E.coli*HbhA * (*p* = 0.0457) and *M. bovis* BCG vs *M. bovis* BCG/r*Rho*HbhA ** (*p* = 0.0127) by one-way ANOVA.

In order to study the interaction of r*E.coli*HbhA and r*Rho*HbhA with epithelial cells, both proteins were incubated with the cells and a pre-absorbed R-anti-HbhA was used to detect the proteins. The binding of both proteins to A549 as detected by fluorescence microscopy was very similar, as shown in Figure 6a.

In order to find out if the presence of either r*E.coli*HbhA or r*Rho*HbhA could have an effect on the internalization of *M. bovis* BCG, epithelial cells pre-incubated with purified recombinant proteins were infected. The results in Figure 6b show that there were no differences in the number of *M. bovis* BCG internalized when A549 cells were pre-incubated with the proteins, but there was a significant inhibition of bacterial entrance with regard to the control without pre-incubation.

### 2.7. rE.coli HbhA and rRhoHbhA Lipid-Binding Assays

As FA are important constituents of triacylglycerol (TAG), they were used as targets for rHbhA. The interaction of r*E.coli*HbhA and r*Rho*HbhA with FA was evaluated by a dot lipid-binding assay. TAG and FA were spotted on PDVF membranes, and stained with phosphomolybdic acid. Glyceryl tristearate and palmitic acid (PA) were not stained and were excluded. From lipids tested for binding to rHbhA, only stearic acid (SA) interacted with both proteins and r*E.coli*HbhA only bound to oleic acid (OA), as shown in Figure S1.

**Figure 7:**
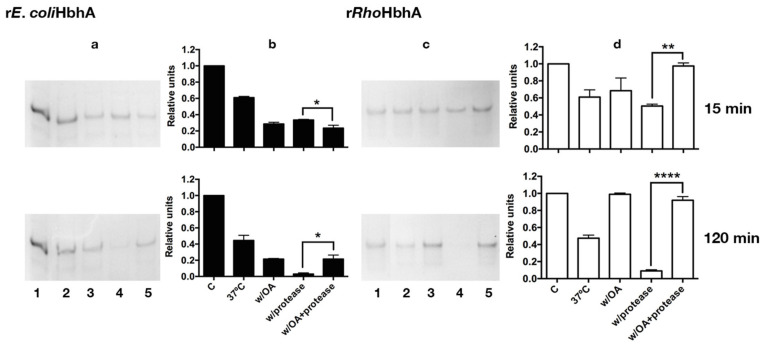
Lipid-binding assay of purified soluble rHbhA. Degradation pattern and stability of apo- and holo-forms of recombinant r*E.coli*HbhA and r*Rho*HbhA, after incubation with and without Glu C for 15 and 120 min. (**a**,**c**) SDS-PAGE, gels were stained with Coomassie blue. Lane 1 (**c**), control protein without any treatment, only one sample was running for both treatment times. Line 2, proteins incubated at 37 °C for 15 and 120 min. Line 3, with oleic acid (W/OA), proteins were incubated with oleic acid at RT for 30 min and then at 37 °C for 15 and 120 min. Line 4, (W/protease), proteins incubated only with Glu C at 37 °C for 15 and 120 min. Line 5, (W/ OA+ protease), proteins incubated with oleic acid, and Glu C at 37 °C for 15 and 120 min. (**b**,**d**) Each protein band was analyzed by using *ImageJ* software. The results represent 2 independent experiments. Significant differences: * (*p* < 0.05), ** (*p* < 0.005) and **** ( *p* < 0.0001).

We also studied the binding to FA of soluble purified r*E.coli*HbhA and r*Rho*HbhA by using a lipid-binding assay that involved the exposition of soluble recombinant proteins and their complexes formed with SA and OA to endoproteinase Glu C, for 15 and 120 min (Figure 7). The band in lane 1 of Figure 7a,c is the same for both treatment times. This band was superimposed in the experiment performed at 120 min, in order to have the same reference of HbhA protein concentration to be analyzed with *ImageJ* software (https://imagej.nih.gov/ij/, accessed on 12 June 2021) (Figure 7b,d).

Samples collected at different times of protease exposition were analyzed by SDS-PAGE. Proteins incubated or not with SA were rapidly degraded by exposition to Glu C independently of treatment, suggesting that in the solution, r*E.coli*HbhA and r*Rho*HbhA did not bind to SA (data not shown). In contrast, specific binding of both recombinant proteins was observed with OA. The protector effect against Glu C activity was clearly seen in the complexes formed between r*E.coli*HbhA or r*Rho*HbhA with OA as shown in Figure 7 (lane 5). Significant differences in protein degradation were observed between the proteins without treatment and those previously incubated with OA.

Interestingly, stability of the apo-forms of both proteins seems to be affected by the incubation at 37 °C. While the holo-forms of methylated r*Rho*HbhA with OA remain relatively stable during the times of treatment independently of the exposition to Glu C, both unmethylated and methylated apo-forms were degraded by activity of the protease.

## 3. Discussion

Lysine methylation of histones in eukaryotes has been considered as one of the most important post-translational modifications, being methylation an epigenetic mark essential for gene regulation and development. Although lysine methylation of non-histone proteins in bacteria is restricted to very few molecules, this modification can play different physiological roles [17]. Even more, methylation of some bacterial proteins is directly related to their immunogenicity and virulence [3,4,8]. 

In mycobacteria, Hlp/HupB is post-translationally modified by lysine acetylation and lysine methylation. Mutation of lysine 86 results in the specific loss of the ability of *Mycobacterium smegmatis* to survive when exposed to isoniazid [7]. 

On the other hand, *M. tuberculosis* HbhA possesses a C-terminal moiety that compromises a heparin-binding domain consisting of lysine-rich repeated motifs that are involved in the ability of bacteria to invade host cells [11]. This domain undergoes mono or di-methylation, containing 20–26 methyl groups on residues 159–199 [6]. Although methylation of HbhA had been associated with the induction of both T and B cell responses [8,18,19,20], the physiopathological role of this modification on the host–pathogen relationship in *tuberculosis* is unknown. 

In this work, *M. tuberculosis hbha* gene was expressed in *E. coli* and *R. erythropolis*, and the latter bacteria were used as alternative host for expression of modified HbhA, based on a previous work in our laboratory where *M. tuberculosis* proteins modified by glycosylation were produced in *R. erythropolis* [21]. The r*Rho*HbhA was expressed as methylated protein in *R. erythropolis* and the presence of methyl groups was evidenced with a R-methylLys-HRP antibody and by mass spectrometry of purified recombinant protein, which showed an increase in MM that could corresponded with the presence of methyl groups. The expression of methylated *M. tuberculosis* HbhA protein in *R. erythropolis* offers some advantages, such as optimization of production time and high level of expression, almost five times higher than the recombinant expressed in *M. smegmatis* [22]. Although it is well known that HbhA is a mycobacterial adhesin that binds to heparan sulfate-containing proteoglycans [5,23], the role of methylation in this interaction has not been established. Previous studies identified the proteoglycans Sdc4 and Sdc1 present in epithelial cells as ligands for HbhA. Surface Plasmon resonance analyses showed that native HbhA expressed in BCG immobilized to Syndecan 1 and 4 showed a K_D_ in the micromolar range (K*_D_* = 1.4 × 10^−5^ Sdc1; K*_D_* = 1 × 10^−5^ Sdc4), suggesting low-affinity interactions between HbhA and both Syndecans [12]. In order to deepen this knowledge, we carried out a comparative ELISA ligand assay using r*E.coli*HbhA and r*Rho*HbhA and heparin bound to streptavidin-coated plates. No differences in heparin binding were found between the unmethylated and methylated proteins. In addition, the affinity for heparin of both unmethylated and methylated rHbhA was also determined by BBI; the affinities were in the micromolar range (K*_D_* = 0.28 μM r*E.coli*HbhA and K*_D_* = 0.68 μM r*Rho*HbhA) with no significant differences between the proteins. Although in this work, small differences were found respect to protein interaction with proteoglycans between methylated and unmethylated proteins, the effect of those apparent small changes could be significant in molecular interactions.

In this line, we studied the entrance of *M. bovis BCG* to A549 epithelial cells previously incubated with either unmethylated or methylated proteins, and no differences were found. However, a significant inhibition of bacterial entrance was observed with regard to the control cells without preincubation with recombinant proteins. Together, these results suggest that the interaction of HbhA with epithelial cells seems to be independent of methylation.

In this work, r*Rho*HbhA was also found in LD induced when recombinant *R. erythropolis* was grown in nitrogen-limited conditions, confirming previous observations demonstrating that HbhA from *M. tuberculosis* was able to bind to LD of the recombinant *M. bovis* BCG strain and *R. opacous* PD63 [16,24]. The localization of this protein in LD had been associated with the assembling and maturation of those structures [14,16]. Biosynthesis and accumulation of TAG and other neutral lipids in LD seems to be a common feature of bacteria belonging to the actinomycetes group, such as *Streptomyces, Rhodococcus* and *Mycobacterium* [25]. *M. tuberculosis* stores FA in the form of TAG under carbon excess and nitrogen starvation [26] and during non-replicative states, such as hypoxia-induced dormancy [27]. LD have also been found in *M. tuberculosis* isolated from the sputum of tuberculosis patients, highlighting the importance of TAG during infection [28]. Importantly, *M. tuberculosis* acquires FA from the human host, which bacteria use to synthesize TAG, and subsequently stored in the form of LD [29]. 

Since the pI is a powerful tool to predict and understand interactions between proteins or proteins and membranes [30], we analyzed this biochemical marker by 2D-E in unmethylated and methylated recombinant proteins. The results were interesting: r*Rho*HbhA showed several spots distributed along the pH gradient, but the majority of them were concentrated in the acid pH region while the spots of unmethylated r*E.coli*HbhA that migrated to the basic pH region, showing lower molecular weight, which could be the result of proteolytic degradation, as has been previously observed for unmethylated HbhA [6]. In fact, the pI of 9.17 predicted for HbhA (ExPASy) corresponds to the pI experimentally determined for native HbhA identified by 2-DE from *M. tuberculosis* extract, obtained from bacteria grown under high iron condition, where this basic spot was identified as HbhA by N-terminal sequence [31]. The presence of methylated spots with low pI in r*Rho*HbhA and r*BCG*HbhA was also confirmed by the recognition of the methyl groups with R-anti-methylLys-HRP. The biochemical consequence of the decreased of pI in methylated HbhA is unknown. It has been reported that one of the most notable characteristics of methylation of some enzymes, such as cytochrome C, is accompanied by lowering the pI. The authors put forward the hypothesis that an altered conformation of apocytochrome c induced by enzymatic methylation would favor its interaction with the receptor on mitochondrial membrane. Those changes only occur in proteins that are transported to intracellular sites, such as cytochrome C [32]. The finding of a putative lipid domain, predicted on the basis of primary amino acid sequence and structure similarity with apolipoprotein E_2_ (PBD:1NFO) and the finding that methylated HbhA bound to 4,5 di-phosphorylated phosphatidylinositol [16], prompted us to use FA as the main constituents of TAG as targets for HbhA. The results showed that by dot lipid-binding assay, both r*E.coli*HbhA and r*Rho*HbhA bound to SA, but only r*E.coli*HbhA was able to bind to OA. Moreover, by using a lipid-binding assay with the soluble forms of both recombinant proteins, it was shown by SDS-PAGE that both proteins bound specifically to OA and the resultant holo-forms were more stable and protected from the enzyme activity. However, higher stability of the complex was observed with methylated protein. Our results suggest that reduction of the pI of HbhA due to methylation could increase the affinity of the protein by FA through an unknown mechanism that would favor a conformational change and stabilization of the protein after its interaction with the FA, allowing them to be safely delivered to LD in benefit of the bacteria.

Further quantitative studies will be necessary to evaluate the role of methylation in stabilization of FA–protein complex and its relationship with the low pI.

Together, these results are showing for the first time that HbhA is a fatty acid binding protein (FABP) that could have an important role in the transport of these molecules to mycobacteria lipids storage compartments. FABP are intracellular molecules mainly identified in eukaryotes. In prokaryotes, they are present only within the order of Actinomycetales. In *M. tuberculosis*, Rv0813c protein was identified as a FABP, on the grounds of its crystal structure. These proteins in bacteria may have roles in the recognition, transport and/or storage of small molecules [33].

The findings obtained in this paper confirm the complexity of HbhA as a moonlighting protein that serves several functions depending on its location [16], and probably on the biochemical changes, such as methylation, that could contribute to the functional complexity of the protein. 

It is worth mentioning that this protein has also been associated with the induction of endoplasmic reticulum stress-mediated apoptosis through the generation of reactive oxygen species and cytosolic Ca2fl in *M. tuberculosis* infected macrophages. The experiments reported were carried out with a recombinant *M. tuberculosis* HbhA methylated expressed in *M. smegmatis* [34]. Since the role of HbHA methylation in ROS generation is unknown, comparative assays with unmethylated protein would be important to define a putative role of this modification in apoptosis and ROS generation.

Furthermore, it is important to mention that although *M. tuberculosis* HbhA has a relevant role in infection and virulence, this protein is also present in non-pathogenic mycobacteria and in actinobacteria. Based on phylogenetic analysis that showed that some regulatory sequences are present only in slow-growing mycobacteria, it has been proposed that the *hbha* gene underwent functional divergence during its evolutionary differentiation, acquiring a new function related to virulence and invasion of host but preserving its ancestral LD binding function, as well as its FA binding capacity, as we found in this work.

In this line, and taking together these observations and our results, it is tempting to propose that methylation of HbhA could be another evolutionary acquisition of mycobacteria that would allow this protein to have more efficient binding to FA, ensuring its transport to cope with mycobacteria nutritional stress and survival. 

Although the differences found in this work regarding protein interaction with proteoglycans and FA between methylated and unmethylated proteins are low, the effect of these apparent small changes in molecular interactions needs to be addressed in further studies. 

Furthermore, our results are showing for the first time that, as occurs in mycobacteria, *Rhodococcus* can also methylate proteins, pointing out that the heterologous production of the recombinant methylated HbhA of *M. tuberculosis* in *R. erytropolis* opens a door to study the role that methylation of this protein has in host–pathogen interaction in tuberculosis. 

## 4. Materials and Methods

### 4.1. Bacterial Strains

*E. coli* TOP10F’, DH5-α, Rosetta (DE3) (Novagen Inc., Madison, WI, USA), *R. erythropolis* strain L88 and *Mycobacterium bovis* BCG Pasteur were used for cloning and expression of *hbha* gene. *E. coli* strains *and R. erythropolis* were grown in LB medium (Difco, Sparks, MD, USA). For *E. coli,* medium was supplemented with 100 μg mL^−1^ of carbenicillin (Invitrogen, Carlsbad, CA USA) (LB/Car) and for *R. erythropolis* with 34 μg mL^−1^ of Chl (Sigma Aldrich, St. Louis, MO, USA). For the induction of LD, *R. erythropolis* recombinants strains were grown in M9G_Hi_N_Low_ [35].

### 4.2. Cloning of M. tuberculosis HbhA Protein in E. coli and R. erythropolis

The coding region of the *hbhA* gene was amplified by PCR with high fidelity DNA polymerase *Pfx* (Invitrogen) from *M. tuberculosis* H37Rv genomic DNA with the oligonucleotide primers *hbhA* 15bF (5′-GCATATGGCTGAAAACTCGAACATTG-3′, NdeI site underlined) and *hbhA* 15bR (5′-CGGATCCTACTTCTGGGTGACCTTCTTGG-3′, BamHI site underlined). The PCR product (600 bp) was cloned into the pCR4 Blunt-TOPO vector (Invitrogen) with the use of TOP10F’ strain. Vector was digested with NdeI and BamHI and the released fragment was gel-purified and ligated into the NdeI and BamHI sites of pET15b (Novagen) and into the thiostrepton (Sigma) inducible pTip-QC1vector (kindly donated by Tomohiro Tamura National Institute of Advanced Industrial Science and Technology, Japan) to generate pET15b-*HbhA* and pTip-QC1-*HbhA,* respectively. The identities and orientation of the inserts were confirmed by restriction analysis and DNA sequencing. 

### 4.3. Expression of M. tuberculosis HbhA in E. coli, Purification as N-Terminal His-Tagged Protein 

*E. coli* Rosetta (DE3) was heat shock-transformed with pET15b-*HbhA*. A single recombinant colony was grown in LB/Car overnight (ON) at 37 °C with shaking (200 rpm). The next day, ON culture was diluted 1/100 in LB/Car and incubated at 37 °C with shaking until OD_600nm_ reached ~0.4. Then, culture was induced with 250 µM (final concentration) of Isopropyl -β-D-1-galactopyranoside (IPTG) (Roche., Applied Science, Mannheim, Germany) and kept for 4 h at 37 °C. Recombinant Histidine-tagged HbhA (r*E.coli*HbhA) was purified from inclusion bodies (IB) by Nickel chromatography, as previously indicated [36].

### 4.4. Expression of M. tuberculosis HbhA in R. erythropolis, Purification as N-Terminal His-Tagged Protein 

pTip-QC1-*HbhA* was used to transform *R. erythropolis* L88 strain. Transformation, induction, expression and lysis were carried out as described before [21]. Briefly, after growth in LB/Chl for 48 h at 26 °C, cells were harvested by centrifugation, re-suspended in lysis buffer (50 mM Tris–HCl, 50 mM NaCl, pH 8.0) and disrupted by sonication during 8 cycles of 1 min of activity and 2 min of rest for 2 h in ice, at 70% of sonicator (Sonics Vibro-cell, Newtown, CN, USA) potency, to obtain the WSE. After centrifugation of this extract, the pellet containing IF was washed twice with 1% Triton X-100 in phosphate buffered saline (PBS) and once with PBS. After that, the sample was solubilized in sample buffer (100 mM Tris-HCl, 50 mM NaCl, 8 M urea, pH 8) and the mixture was stirred ON at room temperature (RT). Protein was purified in an AKTA FPLC system (GE Healthcare Biosciences, Pittsburgh, PA, USA) using a 1 ml Histrap^MT^ column (GE Healthcare), as has been previously described [19]. After washing and equilibration of Ni-NTA column with sample buffer, the sample was loaded and the column was washed with buffer (100 mM Tris-HCl, 50 mM NaCl, 25 mM Imidazole, urea 8 M, pH 8). Then, the protein was eluted with elution buffer (100 mM Tris-HCl, 50 mM NaCl, 500 mM Imidazole, urea 8 M, pH 8.0) at 1 mL/min, by using a gradient (0% to 100% of elution buffer). Fractions displaying the recombinant proteins were pooled and dialyzed against decreasing urea concentrations (from 4, 2, 1 and 0 M). The amount of protein was determined with BCA protein assay kit (Thermo Fisher, Waltham, MA, USA) Proteins were stored at −70 °C until needed. In all the steps, the presence of purified recombinant proteins was monitored by SDS-PAGE.

### 4.5. Molecular Mass Analysis of rRhoHbhA by MALDI-TOF

The molecular mass (MM) of r*Rho*HbhA was determined by MALDI-TOF. A total of 0.8 g of protein was mixed with sinapinic acid saturated with dH_2_O 60%, Acetonitrile 40% and 0.1% trifluoroacetic acid. Mass detection was carried at 1000 to 100,000 Da intervals in microflex^®^ LRF, nitrogen laser spectrometer (Bruker Daltonics, Bremen, Germany) at 337 nm. The parameters used were positive linear mode, 20 KV ionization force and 600 shots. 

### 4.6. Cloning and Expression of M. tuberculosis HbhA Protein in Mycobacterium bovis BCG Pasteur

The *hbhA* gene was amplified from *M. tuberculosis* H37Rv genomic DNA, using Pfu DNA polymerase (Promega., Madison, WC, USA) with primers *hbhA*-5F (5′- GGAGAATTCATGGCAGAAAACTC-3′, EcoRI site in underlined) and *hbhA*-3R (5′-AGCAATACGAGCATGACGGT-3′). 

The 734 bp PCR product was digested with EcoRI and SalI, ligated into the pMV361 vector digested at the same sites. Ligation reactions were transformed into *E. coli* DH5-α and selected in LB with kanamycin (Kan) (MP Biomedicals, Irvine, CA, USA) at 50 μg mL^−1^. pMV361-based plasmids containing *hbhA* were isolated and verified by sequencing, then vector was used to transformed by electroporation into *M. bovis* BCG Pasteur and selected on 7H10/OADC (Difco) agar with 0.5% glycerol and 25 g mL^−1^ of Kan, for 3 weeks at 37 °C. After that, the recombinant mycobacteria were grown in 7H9/ADC (Difco) Kan 20 g mL^−1^ for 28 days. Bacteria were harvested by centrifugation, washed with cold PBS, re-suspended in lysis buffer (50 mM Tris-HCL, 1 mM EDTA, pH 8.0) and disrupted by sonication at 30% of potency, with intervals of 1 min of activity and 1 min of rest for 2 h in ice. Then, the sample was centrifuged at 20,000× *g* for 20 min at 4 °C. The SE was filtered by 0.45 µm and 0.22 µm filter (Corning, Camarillo, CA, USA). To obtain the CW, pellet was washed with PBS, re-suspended and incubated for 2 h at 50 °C in PBS with 2% of SDS. The sample was centrifuged at 20,000× *g* for 15 min at 4 °C. Supernatant was filtered by as described above and proteins were quantified and stored until their use.

### 4.7. Expression of HbhA in R. erythropolis Grown in Low Nitrogen and Carbon Excess Identification of Protein in Purified Lipid Bodies

r*Rho*HbhA and *M. tuberculosis* recombinant glycoproteins PstS-1 (r*Rho*PstS-1) and Apa (r*Rho*Apa) previously expressed in *R. erythropolis* [21] were grown in LB/Chl for 48 h at 26 °C, then cells were harvested by centrifugation and for the induction of LD, they were re-suspended in M9G_Hi_N_Low_. Cultures were induced with 1 µg mL^−1^ thiostrepton and bacteria were grown at 26 °C for an additional 96 h.

After culture centrifugation, cell pellets were either treated as described above to obtain the IF or re-suspended in 25 mM Tricine, 250 mM sucrose, pH 7.8 for isolation of LD by ultracentrifugation, as has been previously described elsewhere, with some modifications [37]. Purified LD were then submitted to different treatments in order to eliminate proteins non-specifically bound [38]. After that, LD were treated with 1 ml of chloroform–acetone (Sigma) (1:1 *v*/*v*) to dissolve lipids and precipitate proteins. The sample was centrifuged at 20,000× *g* for 15 min at 4 °C. Supernatant fractions were dried, and lipids dissolved in chloroform were analyzed by TLC on silica gel 60 plates (Alugram^®^ Xtra SIL G/UV_254_, Macherey-Nagel, Germany) applying the solvent system Hexane/Diethyl ether/Acetic acid 80:20:1. Lipid fractions were visualized by iodine vapor [39]. In this assay, glyceryl trioleate and glyceryl tripalmitate were used as standards. LD proteins and the IF initially obtained were loaded in 12% SDS-PAGE and transferred to PDVF membranes. Membranes were stained with Coomassie blue and proteins were identified by WB with anti-His_6_-HRP (Roche) as described below. 

### 4.8. SDS-PAGE and Two-Dimensional Polyacrylamide Gel Electrophoresis

Ten µg of recombinant extracts, WSE and IF from r*Rho*HbhA, SE and CW from r*BCG*HbhA and 5 µg of purified r*E.coli*HbhA and r*Rho*HbhA from IB and IF, respectively were separated by a 12% SDS-PAGE and transferred to PVDF membranes (Millipore, Burlington, MS USA) that were stained with Coomassie R-250 (Bio-Rad Laboratories, Hercules, CA, USA). For WB, membranes were blocked for 1 h with 3% bovine serum albumin (BSA) (Sigma) in PBS (PBS-BSA) at RT. After that, membranes were incubated 1 h with rabbit anti-HbhA polyclonal antibody (R-anti-HbhA) diluted 1:2000 or with anti-His_6_-HRP diluted 1:2000 in PBS containing 0.05% Tween 20 (PBS-T). After 3 washes with PBS-T, membranes were incubated 1 h with 1:2000 dilution of Protein A-HRP (Invitrogen), washed with PBS-T, and developed with 3 g mL^−1^ of 3,3-diaminobenzidine (DAB) (Sigma) in PBS and 30% hydrogen peroxide (Merck., Darmstadt, Alemania) diluted 1:1000. For detection of methylated lysine, membranes were blocked with 1% skim milk in PBS for 3 h at RT with a wash step with PBS-T, then incubated with rabbit anti-mono/dimethyl lysine horseradish peroxidase antibody (Abcam., Cambridge, UK) (R-methylLys-HRP) diluted 1:1000 in Tris buffer saline (TBS) (50 mM Tris, 150 mM NaCl) with 1% skim milk, ON at 23 °C with shaking. The next day, membranes were washed twice with TBS containing 0.05% Tween 20 and once with only TBS. Buffer was drained and membranes were incubated for a few seconds with SuperSignal West Pico chemiluminescent substrate (Thermo Scientific, Waltham, MA, USA) and developed by using a C-DiGit Blot Scanner (Li-COR, Lincoln, NE, USA).

For 2D-PAGE analysis, isoelectric focusing was performed as described elsewhere, with some modifications [40]. SE from r*BCG*HbhA and purified r*Rho*HbhA and r*E.coli*HbhA from IF and IB, respectively, were desalted in an Illustra NAP 5 column (GE healthcare). Proteins were then concentrated by filtration and precipitated by adding 2% sodium deoxycholate to a final 0.02% concentration, 100% trichloroacetic acid to a final 10% and 200 μL of ice-cold acetone. Protein pellets were re-suspended, and the final volume was adjusted to 125 μL with rehydration buffer (8 M urea, 2% CHAPS, 0.5% IPG buffer pH 4–7, and 20 mM DTT). Approximately 100 µg of SE from r*BCG*HbhA and ~50 µg of recombinant purified proteins were applied on Immobiline DryStrip pH 3–10, 7 cm linear-gradient strips (GE healthcare) for 16 h to dehydrate at room temperature, following the manufacturer’s instructions. Focusing was performed in an Ettan IPGphor 3 Isoelectric Focusing System (GE healthcare) starting at 500 V (for 5 h), increasing potential to 4500 V (90 min) and finally to 14,000 V. After focusing, the strips were equilibrated for 20 min in sample buffer (2% SDS, 50 mM Tris-HCl pH 8.8, 6 M urea, 30% glycerol, 0.002% bromophenol blue, 0.5% DTT). The strips were then loaded onto 12% SDS-PAGE. After electrophoresis, gels were transferred to PVDF membrane stained with Coomassie Brilliant Blue and for antibody detection, membranes were blocked for 1 h with PBS-BSA, and then incubated with R-anti-HbhA or with R-methylLys-HRP and developed as described above.

### 4.9. Binding of rE.coli HbhA and rRhoHbhA to Heparin

The binding kinetics and the determination of the dissociation constant (*K_D_*) for the r*E.coli*HbhA and r*Rho*HbhA against biotinylated heparin (Sigma) was carried out by using Biolayer Interferometry (BLI) at 25 °C. Streptavidin biosensors in an Octet RED96 system (FortéBio Inc., San Jose, CA, USA) were used. The assays were performed on black bottom 96-well microplates (Greiner Bio-One 655209) (Thermo Fisher, Waltham, MA, USA) in a total volume of 200 μL with orbital shaking. Experiments were controlled with the software Data Acquisition 8.2 (ForteBio, Inc., San Jose, CA, USA). For the BLI experiment, a baseline was established using 10× Kinetics buffer (FortéBio Inc., San Jose, CA, USA) diluted to 1× in PBS buffer. Then, the biotinylated heparin at 10 μg mL^−1^ was allowed to bind to streptavidin sensor for 5 min, followed by washing with the same buffer to eliminate nonspecific binding. Next, the r*E.coli*HbhA and/or r*Rho*HbhA were bound to the bound heparin (association). In the last step, a wash (dissociation) was made. The BLI experiment was conducted with five different concentrations of both recombinant proteins. 

New streptavidin biosensors were used for each experiment. The data were processed using the Octet Data Analysis Software version 8.2 (FortéBio Inc., San Jose, CA, USA). 

ELISA was also carried out to evaluate the binding of recombinant proteins to heparin, 0.5 μg/well of biotinylated heparin in PBS was immobilized on streptavidin-coated plates (Thermo Scientific). Samples were incubated ON at 4 °C. The next day, plates were washed twice with PBS-T and then incubated 1 h at RT with 5 μg/well of r*E.coli*HbhA or r*Rho*HbhA diluted in PBS-T/BSA. After 3 washes with PBS-T, wells were incubated for 1 h with R-anti-HbhA diluted 1:2000 and then with Protein A-HRP diluted 1:2000 in PBS-T/BSA. The reaction was revealed with TMB (3,3′,5,5′-tetramethylbenzidine) (Thermo Fisher, Waltham, MA, USA) and stopped with 100 μL of 0.16 M H_2_SO_4_ Absorbance values were measured at OD_450nm_ using an ELISA plate reader (Multiskan Go., Thermo Scientific, Ratastie I, Finland).

### 4.10. Binding of rE.coli HbhA and rRhoHbhA to A549 Epithelial Cells 

To assess the binding of HbhA protein on cells surface, an ex vivo assay was performed using the human lung pneumocyte A549 cell line. The cells were cultured in RPMI 1640 medium, (Gibco, Grand Island, NB, USA) supplemented with 10% heat-inactivated fetal bovine serum (FBS) (Gibco) in 75 cm^2^ Falcon culture flasks (Corning) under standard culture conditions (5% CO_2_ and 37 °C). Then, cells were collected by centrifugation and the viability was determined by Trypan blue exclusion. The incubation with HbhA proteins was performed as described elsewhere [21] with some changes; the cells were seeded at 1.5 × 10^3^ per well in 8-well plates. (Nunc Lab-Tek, Greensburg, PA, USA) in 150 L of RPMI 10% FBS and incubated at 37 °C, 5% CO_2_, for 18 h. After 2 washes with RPMI free of FBS, cells were incubated with r*E.coli*HbhA and r*Rho*HbhA proteins at 26.8 g/L × 10^6^ cells for 2 h at 37 °C with 200 µL of RPMI supplemented with FBS. After 3 washes with PBS-T, cells were fixed for 10 min with 1% Paraformaldehyde, washed 3 times with PBS-T, blocked with PBS-BSA at RT with shaking, and after washing with PBS-T, cells were incubated for 1 h with pre-adsorbed R-anti-HbhA. (R-anti-HbhA serum diluted 1/500 was adsorbed by incubation with 1.5 × 10^3^ A549 cells previously adhered to a slide for 1 h at 37 °C at 5% CO_2_). After 4 washes with PBS-T, cells were incubated with anti-rabbit-AF488 (Invitrogen ) 1/2000 in PBS-BSA for 45 min in agitation and after 3 washes with PBS-T, nuclei were stained with Hoechst (Life Technologies., Bleiswijk, Netherland) 1/9000 for 10 min, then after 3 washes with con PBS-T, cells were covered with a coverslide using vectashield (Vector Laboratories, Burlingame, CA, USA). Fluorescence images were acquired with Olympus BX41 (Fluorescence-microscopy) (Olympus Corporation of the Americas Headquarters, Center Valley, PA, USA) with the 100× magnifying lens, using the program Zen blue 2.6 (Carl Zeiss GmbH, Promenade, Jena, Germany) for capturing digital images.

### 4.11. M. bovis BCG Infection of A549 Cells Previously Incubated with rHbhA Proteins

For infection, A549 cells were pre-incubated with r*E.coli*HbhA and r*Rho*HbhA 10 g/L × 10^6^ cells for 2 h at 37 °C, 5% CO_2_. After that, cells were washed twice with fresh medium and infected with *M. bovis* BCG Danish strain (MOI 3:1) for 1 h in RPMI without FBS and antibiotics. Then, cells were washed 3 times with RPMI and incubated for 1 h in RPMI supplemented with amikacin at 200 μg mL^−1^. Finally, cells were washed and lysed with 100 μL of 0.05% SDS for 1 min and neutralized with 4% BSA. Lysed cells were plated in 7H11/OADC medium. After 21 days of incubation, colony-forming units (CFU) were determined. The assays were performed 3 times. 

### 4.12. Fatty Acid Binding Assays

Dot lipid-binding assay was carried out as described elsewhere [16], with some modifications. Briefly, glyceryl tristearate, glyceryl trioleate, PA (C16:0), SA (C18:0), OA (C18:0) and mineral oil were purchased from Sigma.

PVDF membranes previously wetted in methanol and dried at RT were spotted with 62 μg of each lipid diluted in ethanol while membranes were drying for 1 h at RT in the dark. Lipids were developed with 10% phosphomolybdic acid solution in ethanol, followed by heating to ~60 °C for ~2 min. Only lipids stained with this reagent were used for incubation with rHbhA proteins as follows: membranes were blocked for 1 h in PBS-T/BSA. Then, strips were incubated ON at 4 °C with 5.0 μg mL^−1^ of r*E.coli*HbhA and/or r*Rho*HbhA in PBS-T/BSA with shaking. The next day, after several washes with PBS-T, lipids bound to rHbhA were detected by incubation with R-anti-HbhA 1:3000 for 1 h at RT, followed by incubation with Protein A-HRP (Invitrogen) 1:2000. Strips were revealed with a chemiluminescence substrate of HRP as described above.

We also studied the binding of purified recombinants methylated and un-methylated HbhA proteins to SA and OA by a lipid-binding assay that involved the exposition of soluble recombinant proteins and proteins in complex with FA to endoproteinase Glu C (Promega), which specifically hydrolyzes peptide and ester bonds at the carboxylic side of glutamic acid residues [41], with some modifications. Briefly, samples of 3 μg of r*E.coli*HbhA and r*Rho*HbhA were incubated for 30 min at RT with OA or SA dissolved in ethanol at a ratio of 1:4 (protein:FA) in buffer 20 mM Tris–HCl, 50 mM NaCl, 1 mM DTT. After that, Glu C was added at a radio of 1:20 (enzyme:protein) and further incubated at 37 °C for 15 and 120 min. As controls, recombinant proteins without any treatment or treated with Glu C as well as proteins in complex with FA but without Glu C treatment were incubated at 37 °C for 15 and 120 min. After different times of treatment, samples were boiled in sample buffer and loaded in 12% SDS-PAGE. Gels were stained with Coomassie blue. 

### 4.13. Statistical Analysis

GraphPad Prims version 6.0 software was used to analyze the results shown in Figure 5, Figure 6 and Figure 7. For the statistical analyses, one-way ANOVA multiple comparations with Tukey’s correction was used. 

## Data Availability

The data presented in this study are available on request from the corresponding author.

## Supplementary Materials

The following are available online at https://www.mdpi.com/article/10.3390/pathogens10091139/s1. Figure S1: Dot Lipid-binding assays with TAG, glyceril trioleate (line 1) stearic acid (line 2), oleic acid, (line 3) and mineral oil (line 4) were spotted on PVDF membranes and incubated with rE.coliHbhA and rRhoHbhA. In control row, rHbhA proteins were omitted. Control of rHbhA protein used for the assay spotted to membrane and developed with R-antiHbhA is also shown. Phosphomolybdic acid stain was carried out to confirm the presence of lipids on membrane.
