# Supplementary material for: New Insights into the Methylation of Mycobacterium tuberculosis Heparin Binding Hemagglutinin Adhesin Expressed in Rhodococcus erythropolis
Source: Pathogens. 2021 Sep 4;10(9):1139. doi: 10.3390/pathogens10091139 (PMC8467707; doi:10.3390/pathogens10091139)
Supplement: Supplementary file 1 [file pathogens-10-01139-s001.zip › pathogens-1324920-supplementary.pdf]

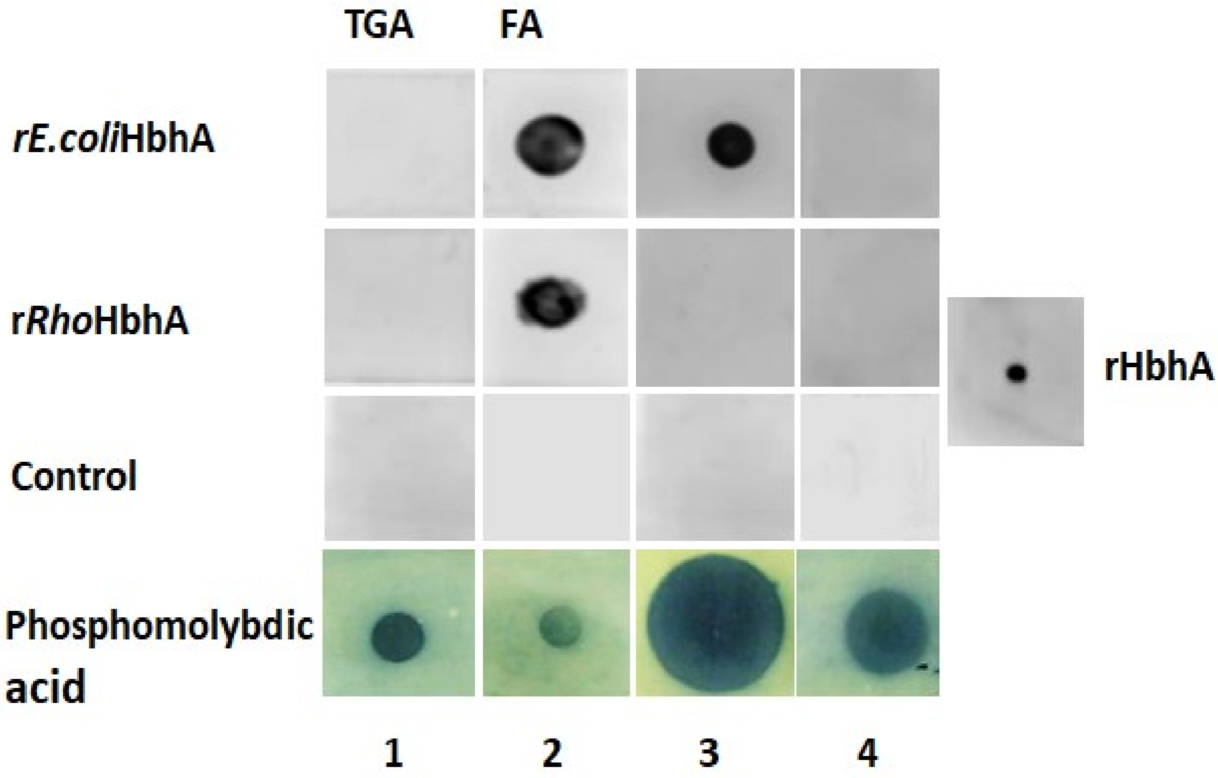

Figure S1. Dot Lipid-binding assays with TAG, glyceril trioleate (line 1) stearic acid (line 2), oleic acid, (line 3) and mineral oil (line 4) were spotted on PVDF membranes and incubated with r*E.coli*HbhA and r*Rho*HbhA. In control row, rHbhA proteins were omitted. Control of rHbhA protein used for the assay spotted to membrane and developed with R-antiHbhA is also shown. Phosphomolybdic acid stain was carried out to confirm the presence of lipids on membrane.
